# New Fault Recognition Method for Rotary Machinery Based on Information Entropy and a Probabilistic Neural Network

**DOI:** 10.3390/s18020337

**Published:** 2018-01-24

**Authors:** Quansheng Jiang, Yehu Shen, Hua Li, Fengyu Xu

**Affiliations:** 1 School of Mechanical Engineering, Suzhou University of Science and Technology, Suzhou 215009, China; yehushen@mail.usts.edu.cn (Y.S.); lihua@mail.usts.edu.cn (H.L.); 2 Suzhou Key Laboratory of Precision and Efficient Machining Technology, Suzhou 215009, China; 3 College of Automation, Nanjing University of Posts and Telecommunications, Nanjing 210003, China; xufengyu598@163.com

**Keywords:** fault recognition, information entropy, probabilistic neural network, rotary machinery, feature extraction

## Abstract

Feature recognition and fault diagnosis plays an important role in equipment safety and stable operation of rotating machinery. In order to cope with the complexity problem of the vibration signal of rotating machinery, a feature fusion model based on information entropy and probabilistic neural network is proposed in this paper. The new method first uses information entropy theory to extract three kinds of characteristics entropy in vibration signals, namely, singular spectrum entropy, power spectrum entropy, and approximate entropy. Then the feature fusion model is constructed to classify and diagnose the fault signals. The proposed approach can combine comprehensive information from different aspects and is more sensitive to the fault features. The experimental results on simulated fault signals verified better performances of our proposed approach. In real two-span rotor data, the fault detection accuracy of the new method is more than 10% higher compared with the methods using three kinds of information entropy separately. The new approach is proved to be an effective fault recognition method for rotating machinery.

## 1. Introduction

Rotating machinery plays a key role in industrial production. Once failure occurs, it may lead to significant downtime losses. The condition monitoring and fault identification diagnosis for rotating machinery is an important guarantee to improve the reliability of mechanical equipment operations [1]. Since the fault feature information of rotating machinery is complex and changeable, it is important for mechanical fault diagnosis to accurately extract the intrinsic characteristics from all kinds of fault signals [2].

Common signal feature extraction methods for rotating machinery include time domain and frequency domain methods [3], e.g., time domain analysis, spectrum analysis, correlation analysis, zoom spectral analysis, independent component analysis, wavelet transform, as well as empirical mode decomposition [4,5,6]. These methods are effective only for single fault diagnosis.

As modern rotating machinery becomes more complex and intelligent, the state features have non-stationary dynamic and multi-source coupling characteristics [7]. There are many factors that cause faults. A new fast estimator of the spectral correlation (the fast spectral correlation) based on the short-time Fourier transform (STFT) for a cyclostationary signal is proposed [8]. One kind of fault can be described with different characteristic indices. The same symptom is often the result of several faults, and the fault feature and, as a result, the mapping between the fault feature and the fault source is nonlinear [9]. The complexity of fault diversity, uncertainty, and the connection between various faults constitute the technical difficulties for fault diagnosis. It is difficult to extract effective fault feature information and diagnose faults by means of a single fault feature and diagnosis method. Therefore, it is necessary to use information fusion methods to combine multi-symptom information.

Entropy is a physical quantity that represents the degree of regularity and complexity of the system. It has been applied to the field of mechanical fault diagnosis in recent years [10,11,12]. Antoni [13] proposed an entropic evidence method to measure the negentropy of the squared envelope (SE) and of the squared envelope spectrum (SES) of the signal, which can capture the signature of repetitive transients in time or frequency domains. Gao et al. [14] proposed an ensemble empirical mode decomposition (EEMD) method-based singular spectrum entropy for signal analysis and fault diagnosis of rotating machinery. A fault detection method based on multi-sensor data fusion for planetary gearboxes is presented in [15]. Compared with the methods based on individual sensors, the proposed method achieves much higher accuracies in detecting planetary gearbox faults. Bai et al. [16] presented a fault quantitative diagnosis method which described the change disciplinarian of rotor vibration by the power spectrum entropy with more measurement points and multiple speeds based on information entropy theory. Xu et al. [17] used approximate entropy to describe the irregularity and complexity of mechanical vibration signals and measured the complexity of two-dimensional signals of axis orbits. It also quantitatively evaluated the running status of rotating machinery. An approximate entropy-based method for machine health monitoring from a rolling bearing is proposed in [18]. Gui et al. [19] proposed wavelet packet characteristic entropy and a neural network for dynamic feature extraction and fault detection of a pressure pulsation signal of a turbine draft tube. When a rolling bearing is at the early fault stage, the signal is easily contaminated by the gear and other noise sources, and Sun et al. [20] presented a method of singular point recognition and feature extraction for the incipient bearing fault based on instantaneous envelope scalogram entropy. Jing [21] proposed an adaptive multi-sensor data fusion method based on deep convolutional neural networks (DCNN) for fault diagnosis. It obtained satisfactory diagnosis accuracy.

Staszewski [22] proposed a method of wavelet-based compression and feature selection for periodic, continuous, non-stationary, and transient non-stationary signals analysis. Kahirdeh et al. [23] utilized acoustic entropy to characterize the Materials in the Course of Degradation, and demonstrated that the maximum acoustic entropy that can emanate from materials during the course of degradation remains similar. Cui et al. [24] proposed a method of compressor valve fault diagnosis using information entropy and an SVM, obtaining good performance from experiments. Cabal et al. [25] presented a methodology for induction motor condition monitoring by analyzing a single parameter, which can detect different faults quantitatively.

As an intelligent data processing method, neural networks have a strong ability to deal with the nonlinear characteristics in the data. Among them, a probabilistic neural network (PNN) can approximate any nonlinear continuous function with arbitrary precision, and has the ability of self-organization, self-learning, fast learning speed, and parallel processing [26,27,28]. Deep learning is one of the hotspots in artificial intelligence (AI) techniques [29]. A novel AI method based on a deep belief network (DBN) is proposed for the unsupervised fault diagnosis of a gear transmission chain in [30].

In this paper, for the purpose of quantifying the characteristics of vibration signals, we propose a feature information fusion model based on a probabilistic neural network and information entropy. We extract three kinds of information entropy of vibration signals in the time domain, frequency domain, and signal complexity, respectively, according to information theory. We also conduct a typical fault diagnosis verification experiment for rotating machinery. The remainder of the paper is organized as follows: Section 2 describes three kinds of information entropy features. Then the probabilistic neural network is discussed in Section 3. The information entropy features fusion model is designed and analyzed in Section 4. The simulation and application experiments analysis for the presented features fusion model are carried out in Section 5. Conclusions of this paper are presented in Section 6.

## 2. Descriptions of Information Entropy Features

Information entropy is a description of the degree of uncertainty of the system, so we can use it to measure the state change of the rotating machinery. A lower entropy value means less uncertainty of the information. That is to say, there are fewer disorders in the information.

The definition of the entropy within the system is as follows [31]:

Assume *M* is a Lebesgue space which is a σ-algebra and generated by measurable set *S*. It has *μ* measure and *μ*(*M*) = 1. *M* could be described by a finite partition $A=\{A_{i}\}$ which is incompatible. That is to say: $M=\overset{n}{\underset{i=1}{\cup }}A_{i}$ and $A_{i}\cap A_{j}=\Phi ,\forall i\neq j$. Entropy with regard to partition A is defined as:

(1) $$H(A)=-\sum_{i=1}^{n}\mu (A_{i})\log\mu (A_{i})$$

where $\mu (A_{i})(i=1,2,3,\cdots n)$ is the measure for set $A_{i}$.

According to the theory of entropy, we analyze the energy features of vibration signals in the time and frequency domains. Furthermore, we extract entropy features in vibration signals and generate the following three kinds of features.

### 2.1. Singular Spectrum Entropy

Singular spectral entropy gives an indicator to measure the complexity or uncertainty of a vibrational signal with multiple spatial distributions on the whole. For a discrete time series $Y_{t}=[y_{1},y_{2},\cdots y_{N}]$ (*N* is the number of samples) of the vibration signal, we can map the original signal into the embedding space by using the delay inlay technique. Assuming that the length of the embedding is *M*, we can obtain an (N−M+1)×M dimensional matrix:

(2) $$A=\left[\begin{array}{cccc} y_{1} & y_{2} & \cdots & y_{M}\\ y_{2} & y_{3} & \cdots & y_{M+1}\\ \vdots & \vdots & \vdots & \vdots \\ y_{N-M} & y_{N-M+1} & y_{N-M+2} & y_{N} \end{array}\right]$$

The singular values can be obtained by the SVD decomposition from the matrix *A*. The definition of singular value decomposition is as follows [32]:

We define *Y* as a matrix with the dimension m×n, there are two orthogonal matrices $U=[u_{1},\cdots u_{m}]\in R^{m\times m}$, $V=[v_{1},\cdots v_{n}]\in R^{n\times n}$ which satisfy the following equation:

(3) $$Y=UDV^{T},\;D=diag(\sigma_{1},\cdots \sigma_{r})\in R^{m\times n},\;r=rank(Y)$$

$\sigma_{i}=\sqrt{\lambda_{i}}(i=1,2,\cdots ,r,\cdots ,m)$ can be called the singular value of the matrix *Y*, where $\lambda_{1}\geq \lambda_{2}\geq \cdots \geq \lambda_{r}\geq 0,\;\cdots ,\lambda_{r+1}=\lambda_{r+2}=\cdots =\lambda_{m}=0$.

Thus, we decompose *A* with the SVD decomposition, and obtain all of the singular values $\left[\sigma_{1},\sigma_{2},\cdots \sigma_{m}\right]$. All of the singular values form the singular spectrum entropy of the vibration signal. We assume *k* is the number of non-zero singular values, and *k* stands for the number of different patterns of the column space of matrix *A*. Singular spectrum entropy can be defined as follows:

(4) $$H_{s}=-\sum_{i=1}^{m}p_{i}\log p_{i}$$

where $p_{i}=\frac{\sigma_{i}}{\sum_{i=1}^{m}\sigma_{i}}$ is the relative weight of the *i*th singular value with regard to all the singular values.

Singular spectrum entropy extracts the intrinsic complexities of the system and describes its status species according to the SVD technology.

### 2.2. Power Spectrum Entropy

We assume Y(ω) as the discrete Fourier transform of the vibration signal $Y_{t}=[y_{1},y_{2},\cdots y_{N}]$ (*N* is the number of samples). Then the power spectrum of $Y_{t}$ is $S(\omega )=\frac{1}{2\pi N}{\left|Y(\omega )\right|}^{2}$ [33]. According to the energy conservation law, we set:

(5) $$\sum y^{2}(t)\Delta t=\sum {\left|Y(\omega )\right|}^{2}\Delta \omega$$

As a result, the power spectrum $S=\left\{S_{1},S_{2},\cdots S_{n}\right\}$ could be considered as a partition of the vibration signal in frequency domain. Thus, we can define the corresponding power spectrum entropy as follows.

(6) $$H_{f}=-\sum_{i=1}^{n}q_{i}\log q_{i}$$

where $q_{i}=\frac{S_{i}}{\sum_{i=1}^{n}S_{i}}$ is the proportion of the *i*th power spectrum with regard to the whole power spectrum.

From the definition of the power spectrum entropy, we know that the power spectrum entropy of the vibration signal depicts the distribution modes of the vibration energy in the frequency domain.

### 2.3. Approximate Entropy

Approximate entropy is a new metric to evaluate the complexity of a series. It estimates the probability of new mode generation according to the analysis of the complexity of a time series [34]. A non-negative value is applied to describe this complexity. We show an approximate entropy algorithm as follows:

We assume {u(i)}(i=1,2,3⋯N) (*N* is the number of samples) is the given time series, *m* is the predefined number of dimensions of the patterns, and *r* is the predefined similarity threshold. The algorithm is as follows:

(1) Form the series of data {u(i)}(i=1,2,3⋯N) into an *m* dimensional vector X(i);
(2) Calculate the distance between X(i) and X(j):
  $$d[X(i),X(j)]=\underset{k=0:m-1}{\max}\left|u(i+k)-u(j+k)\right|\left(i=1:N-m+1,j=1:N-m+1,i\neq j\right)$$
(3) Count the number of distances that satisfy d[X(i),X(j)]<r and set it as num. Then we can calculate the ratio between *num* and the total number of vectors as follows:
  (7) $$C_{i}^{m}(r)=\{d[X(i),X(j)]<r\}/(N-m+1)$$
(4) Take the logarithm for each $C_{i}^{m}(r)$ and obtain their mean:
  (8) $$\phi^{m}(r)=\frac{1}{N-m+1}\sum_{i=1}^{N-m+1}\ln C_{i}^{m}(r)$$
(5) Set the dimension to be m + 1 and repeat the procedures from Step 1 to Step 4. We can obtain $\phi^{m+1}(r)$.
(6) The approximate entropy of the series {u(i)}(i=1,2,3⋯N) can be calculated with the following equation:
  (9) $$ApEn(m,r,N)=\phi^{m}(r)-\phi^{m+1}(r)$$

According to the definition of approximate entropy, we know that it is a function with respect to the dimension of the pattern, similarity threshold, and the number of samples. It estimates the probability of new pattern generation for a time series when the dimension changes. It is an effective non-linear analysis method since it only needs data with small length and could be applied to both determined and stochastic processes.

## 3. Probabilistic Neural Network (PNN)

The basic idea of a probabilistic neural network is to generate a decision space in a high-dimensional input space based on Bayesian rules, which is to say to minimize the expected risk of erroneous classification. A probabilistic neural network is a kind of artificial neural network which is based on statistics. More specifically, it is a kind of feed-forward network whose activation function is the Parzen window function. A PNN contains the strengths of both the Radial Basis Function (RBF) neural network and the classical probabilistic density estimation method. It performs better in pattern classification compared with traditional feed-forward neural networks.

**Figure 1:**
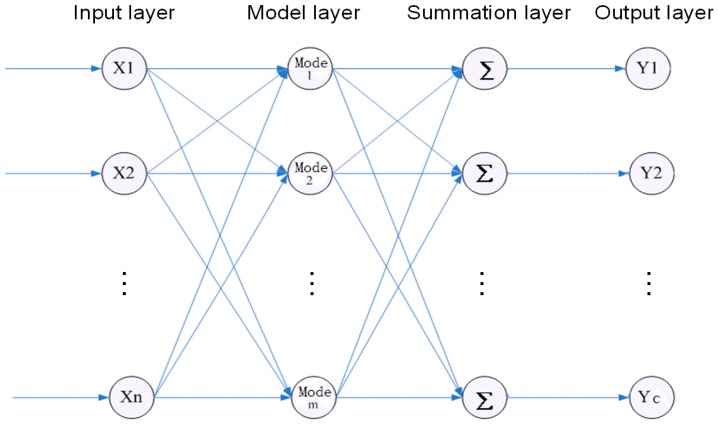
Structure diagram of the PNN.

We map the sample space to pattern space with the help of the PNN. As a result, we can obtain a network system which is robust and has an adaptive structuring ability. The structure of the PNN is similar with that of an RBF neural network. As shown in Figure 1, the main structure contains four layers: input layer, model layer, summation layer, and output layer.

The input layer receives the samples (the input dimension is *n*). The model layer is responsible for calculating the pattern correspondence between the input feature vector and the training set. It will generate a non-linear function, i.e., $\exp[(n_{k}-1)/\sigma^{2}],\;(n_{k}=w_{k}^{T}x)$. Here $w_{k}$ is the connection weight between the input layer and the model layer. We can obtain the distance $p_{j}^{i}$(i=1,2,⋯,m, where m is the number of models in the sample) between the input vector and the weight vector from every model layer unit. Finally, we can obtain the conditional probabilistic density in the summation layer:

(10) $$P_{k}=\sum_{j=1}^{m_{k}}\pi_{j}^{i}p_{j}^{i}$$

The output layer estimates the maximum probability $R_{k}$ of a test sample, which belongs to a specific class based on Bayesian minimal risk estimation theory:

(11) $$R_{k}=\sum_{l=1}^{c}v_{l}^{k}\alpha_{l}P_{l}$$

Thus, we can obtain the expected class of the test sample.

Since the weights between the input layer and the model layer could be adaptively chosen according to the training samples, the PNN is simple, robust, and easy to train.

## 4. Information Entropy Features Fusion Model

Information fusion is a signal processing procedure. It could manipulate complex multi-source information from different scales and aspects.

The fusion process could be divided into three levels according to the relationships among multi-source information: data-level fusion, feature-level fusion, and decision-level fusion. The main procedures for feature-level fusion are as follows: Firstly, we transform the raw data from every sensor into a feature vector. Then we fuse all these feature vectors. We obtain our final decision based on the fused results. The fused results compressed the raw data and extracted the key information. As a result, it could reduce the computational complexity.

**Figure 2:**
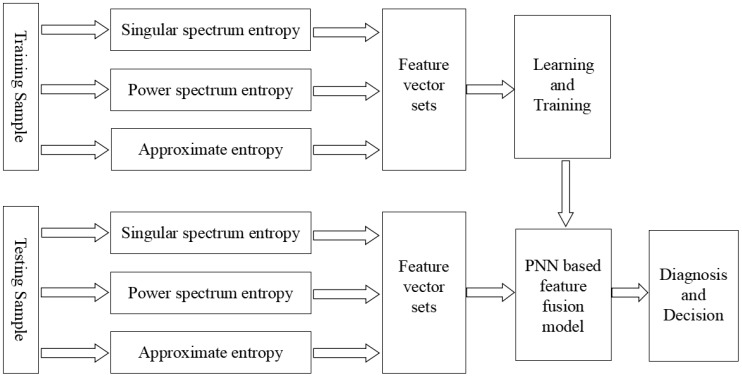
The structure of the feature fusion model based on information entropy and the PNN.

This paper proposes a feature fusion model based on information entropy and probabilistic neural networks for rotary machine fault identification. The structure of the fusion model is demonstrated in Figure 2.

According to Figure 2, the main steps of the feature fusion model based on information entropy and the PNN are as follows.

(1) The input data are collected from all the sensor outputs for fault identification. We calculated singular spectrum entropy, power spectrum entropy, and approximate entropy from the training data and construct the feature vectors. We feed the feature vectors into the PNN as training samples and train the model based on these samples.
(2) We calculate the corresponding feature vector for each test sample and feed the vector to the PNN which has already trained in Step 1. We can obtain the fault identification result from the output layer of PNN.
(3) For each test sample output, if its classification result is correct, we could add it into the training set. We retrain the whole model with this new training set in order to enhance the performance of the model.

## 5. Simulation and Application Experiments

### 5.1. Simulated Fault Signal

In this section, we generated three kinds of simulated fault signal of rotary machines as training and test samples.

For all the simulated signals, Class A stands for the type of unbalanced fault, Class B stands for the type of coupling misalignment fault, Class C stands for the type of rubbing fault. We set the sampling number to be 1024 and the rotation speed of the rotor is 3000 rpm. Thus, the working frequency of the rotor is $f_{1}=50\;\mathrm{Hz}$, double the fundamental frequency is $f_{2}=100\;\mathrm{Hz}$, high frequency is $f_{3}=200\;\mathrm{Hz}$, half the fundamental frequency is $f_{4}=25\;\mathrm{Hz}$, and extremely high frequency is $f_{4}=500\;\mathrm{Hz}$. We construct the simulated signals according to the different constitutions of frequencies for different faults.

Class A consists of 90% of frequency *f*_1_, and 5% of frequency *f*_2_ and *f*_3_, respectively. Thus, the signal can be represented as follows:

(12) $$X_{1}(t)=0.9\cos(2\pi f_{1}t)+0.05\cos(2\pi f_{2}t)+0.05\cos(2\pi f_{3}t)+\varepsilon (t)$$

where *ε*(*t*) is Gaussian noise whose mean is 0 and standard deviation *σ* is 0.1.

Class B consists of 50% of frequency *f*_2_, 40% of frequency *f*_1_, and 10% of frequency *f*_3_. Thus, the signal can be represented as follows:

(13) $$X_{2}(t)=0.4\cos(2\pi f_{1}t)+0.5\cos(2\pi f_{2}t)+0.1\cos(2\pi f_{3}t)+\varepsilon (t)$$

Class C consists of 40% of frequency *f*_1_, 20% of frequency *f*_2_, 10% of frequency *f*_3_, 20% of frequency *f*_4_, and 10% of frequency *f*_5_. Thus, the signal could be represented as follows:

(14) $$X_{2}(t)=0.4\cos(2\pi f_{1}t)+0.2\cos(2\pi f_{2}t)+0.1\cos(2\pi f_{3}t)+0.2\cos(2\pi f_{4}t)+0.1\cos(2\pi f_{5}t)+\varepsilon (t)$$

In Equations (12)–(14), *ε(t)* is Gaussian white noise whose mean is 0 and standard deviation *σ* is 0.1.

We generate 100 groups of data from the above-mentioned three classes, respectively. We calculate the singular spectrum entropy, power spectrum entropy, and approximate entropy, respectively, from the training data and form them into feature vectors.

We randomly split the generated data into two halves. We use one half as the training set and train the PNN. The other half is used as the test set. Table 1 compares the results of the four methods. The first three methods use singular spectrum entropy, power spectrum entropy, and approximate entropy separately. The last method fuses all three kinds of entropies with the PNN.

From Table 1 we can conclude that the fault identification accuracy becomes much higher while fusing all three entropies when compared to using the three kinds of information entropy separately. This reflects the better performances and effectiveness of the proposed method based on information entropy and a probabilistic neural network in fault diagnosis.

### 5.2. Rotor Test Platform Fault Experiments

In this section, we collect signals from the rotor test platform, and calculate three kinds of information entropy from the collected signals.

The used rotor test rig is an experimental device to simulate the vibration condition of rotating machinery, which can effectively reproduce many kinds of vibration phenomena generated by rotating machinery. The rotor rig can simulate the running state of the machine by changing the rotor speed, shaft stiffness, mass unbalance, bearing friction, coupling form, or impact condition through different choices.

**Figure 3:**
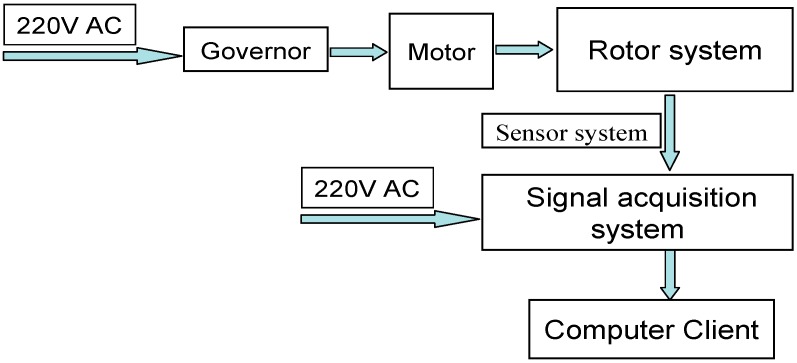
The working principle diagram of the rotor test rig.

The working principle diagram of the rotor test rig is shown in Figure 3.

We conduct experiments for identifying unbalance fault, coupling misalignment fault and rubbing fault with the propose feature fusion model.

#### 5.2.1. Identifying Unbalance and Rubbing Faults for a Single-Span Rotor

We design a PNN model with three input units and two output units. The three input units correspond to the above-mentioned three kinds of information entropy and the two output units correspond to unbalance and rubbing faults, respectively.

We tested at three different rotation speeds, which are 1200 rpm, 2400 rpm, and 3600 rpm, for a single-span rotor. We collected vibration fault signals at the three rotation speeds. For each rotation speed, we collected 100 groups of data for training and 20 groups of data for testing.

**Figure 4:**
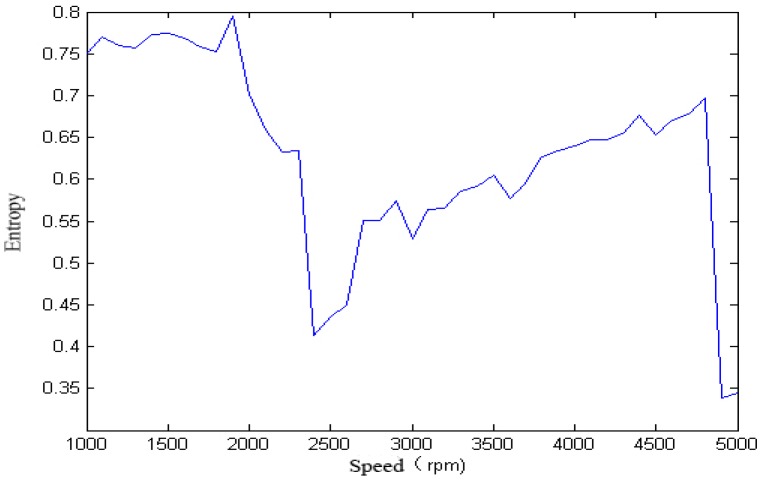
Singular spectrum entropy of a single-span rotor under the unbalance fault.

**Figure 5:**
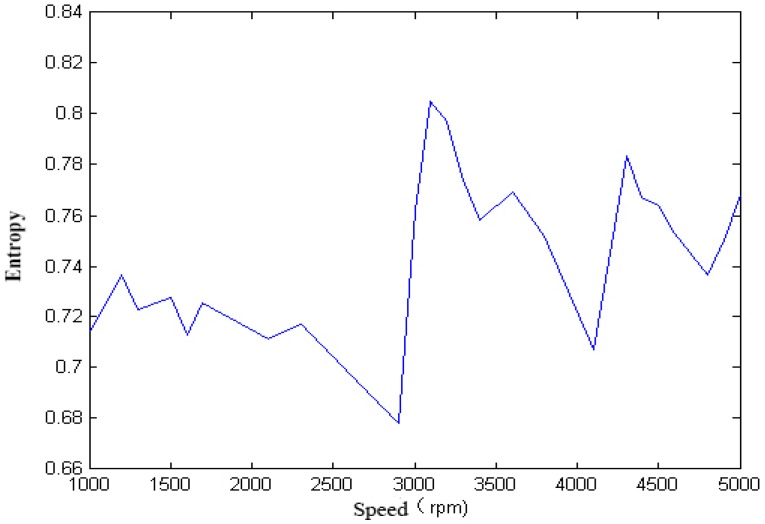
Singular spectrum entropy of a single-span rotor under the rubbing fault.

For the single-span rotor, the calculated singular spectrum entropy of each vibration signal in different rotation speeds is shown in Figure 4 and Figure 5.

**Figure 6:**
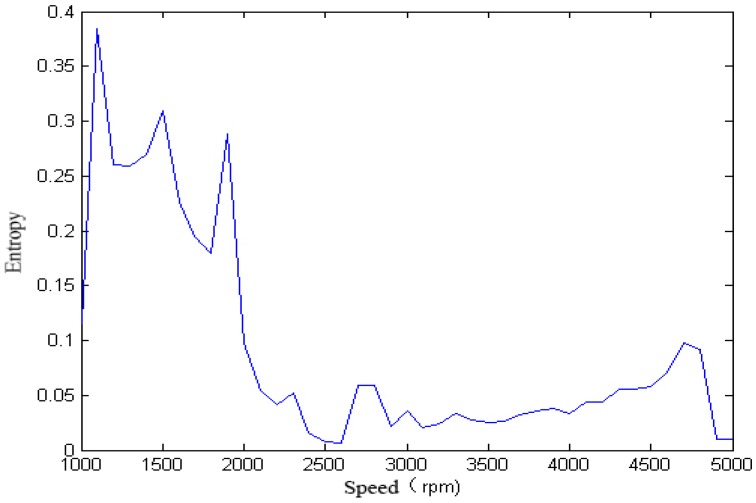
Power spectrum entropy of a single-span rotor under the unbalance fault.

**Figure 7:**
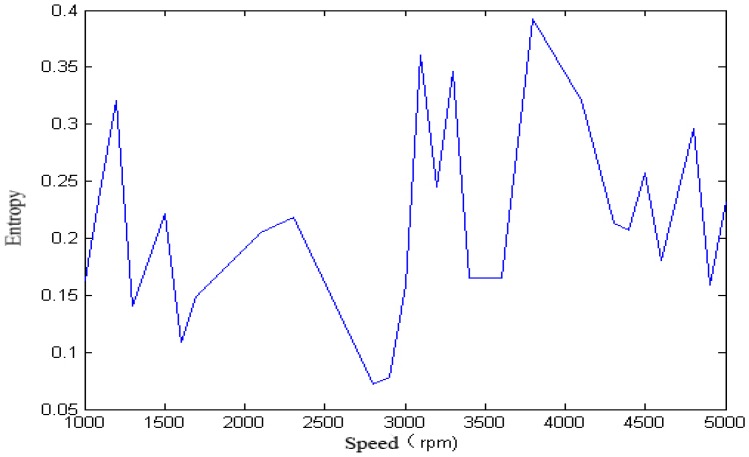
Power spectrum entropy of a single-span rotor under the rubbing fault.

We calculated the singular spectrum entropy of each vibration signal in the single-span rotor at different rotation speeds, and the result is shown in Figure 6 and Figure 7.

**Figure 8:**
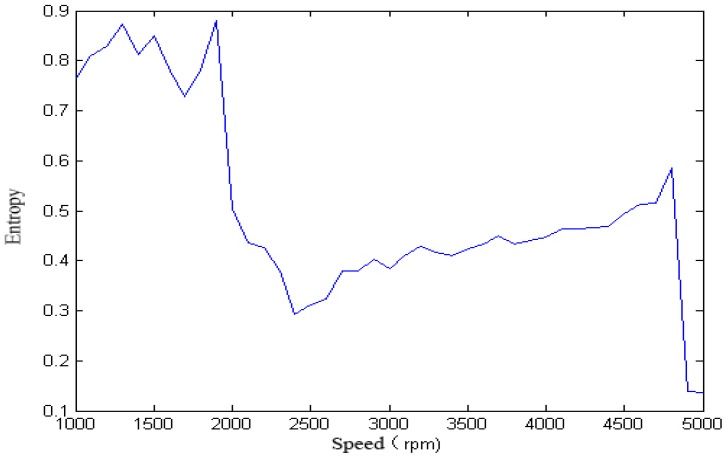
Approximate entropy of a single-span rotor under the unbalance fault.

**Figure 9:**
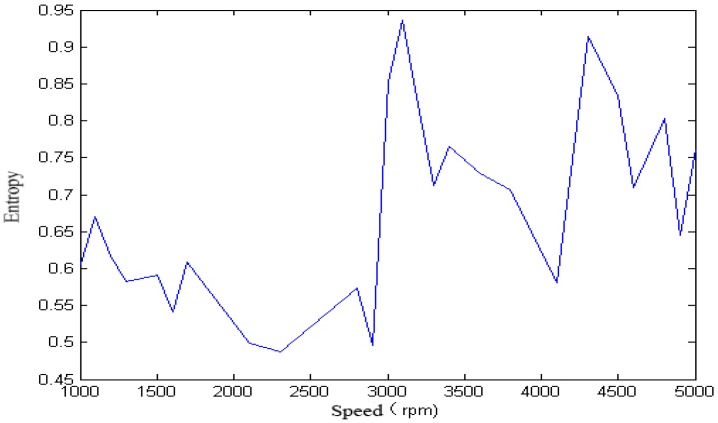
Approximate entropy of a single-span rotor under the rubbing fault.

We calculated the approximate entropy of each vibration signal in the single-span rotor at different rotation speeds, and the result is shown in Figure 8 and Figure 9.

It can be seen from Figure 5, Figure 6, Figure 7, Figure 8 and Figure 9 that, with the change of speed, the variation law of entropy of a single-span rotor in different failure states presents different trends, and the range of variation is wide. With the increase of speed, the singular spectrum entropy, power spectrum entropy, and approximate entropy in the unbalance fault show a generally decreasing trend, while the singular spectrum entropy, power spectrum entropy, and approximate entropy of the rubbing fault show a generally increasing trend. Table 2 compares the fault identification accuracy rate of the four methods.

From Table 2 we can conclude that there is no clear winner for any single kind of entropy from singular spectrum entropy, power spectrum entropy, and approximate entropy. Their accuracies are much lower than that of using the feature fusion model. This is because the above three kinds of entropy describe the characteristics of signals from the time domain, frequency domain, and complexity domains, respectively. The proposed feature fusion model could combine information from different characteristics and, as a result, it is more sensitive to the fault features and could boost the performance.

#### 5.2.2. Identifying Unbalance, Coupling Misalignment Faults, and Rubbing Faults for Two-Span Rotors

We designed a PNN model with three input units and three output units. The three input units correspond to the above-mentioned three kinds of information entropy and the three output units correspond to the unbalance, coupling misalignment fault and rubbing faults, respectively.

Similar with the above experiment, we test at three different rotation speeds, which are 1000 rpm, 3000 rpm, and 5000 rpm. We collected vibration fault signals at the three rotation speeds. For each rotation speed, we collected 100 groups of data for training and 20 groups of data for testing. Table 3 compares the results of the four methods.

From Table 3 we can see that, when the singular spectrum entropy, power spectrum entropy, and approximate entropy are used separately to classify the faults of two-span rotors, the classification accuracy is not satisfactory, and the fault characteristics cannot be better reflected. However, for the proposed feature fusion model method, the fault classification accuracy is significantly higher than that of any single feature fault classification methods. This proves the effectiveness of the proposed approach.

## 6. Conclusions

In order to cope with the difficulty of identifying complex faults for rotary machinery, this paper proposes a feature fusion model based on information entropy and a probability neural network. In order to obtain the final fault identification result, we implement the characteristic measurement of a vibration signal combining singular spectrum entropy, power spectrum entropy, and approximate entropy, and fuse them with a PNN model to classify and diagnose the fault signals. Finally, the fault detection accuracy of our proposed method is more than 10% higher compared with the methods using three kinds of information entropy separately in real two-span rotor data, and proved to be an effective fault diagnosis method.

## Figures and Tables

**Table 1:** Results of four fault identification methods on simulated data.

Methods	Singular Spectrum Entropy	Power Spectrum Entropy	Approximate Entropy	Feature Fusion Model
Fault identification accuracy	91.7%	93.5%	68.3%	95.2%

**Table 2:** Results of four fault identification methods on real data of a single-span rotor.

Rotation Speed	Singular Spectrum Entropy	Power Spectrum Entropy	Approximate Entropy	Feature Fusion Model
1200 rpm	71.2%	78.9%	78.9%	83.1%
2400 rpm	81.6%	92.2%	86.9%	94.8%
3600 rpm	93.9%	64.3%	96.2%	98.7%

**Table 3:** Results of four fault identification methods on real data of two-span rotors.

Rotation Speed	Singular Spectrum Entropy	Power Spectrum Entropy	Approximate Entropy	Feature Fusion Model
1000 rpm	67.74%	64.51%	66.13%	80.64%
3000 rpm	73.69%	81.03%	79.52%	92.17%
5000 rpm	82.85%	75.97%	85.21%	95.38%
